# Glymphatic system function in patients with newly diagnosed focal epilepsy

**DOI:** 10.1002/brb3.2504

**Published:** 2022-02-02

**Authors:** Dong Ah Lee, Bong Soo Park, Junghae Ko, Si hyung Park, Jin‐Han Park, Il Hwan Kim, Yoo Jin Lee, Kang Min Park

**Affiliations:** ^1^ Department of Neurology, Haeundae Paik Hospital Inje University College of Medicine Busan Korea; ^2^ Department of Internal Medicine, Haeundae Paik Hospital Inje University College of Medicine Busan Korea

**Keywords:** diffusion tensor imaging, epileptic seizure, glymphatic system

## Abstract

**Introduction:**

The aim of this study was to analyze the glymphatic system function and its relationship with clinical characteristics, global diffusion tensor imaging (DTI) parameters, and global structural connectivity in treatment‐naïve patients with newly diagnosed focal epilepsy.

**Methods:**

This retrospective single‐center study investigated patients with focal epilepsy and healthy controls. All participants underwent routine brain magnetic resonance imaging and DTI. DTI analysis along the perivascular space (DTI‐ALPS) was used to evaluate glymphatic system function. We also calculated the measures of global DTI parameters, including whole‐brain fractional anisotropy (FA), mean diffusivity (MD), axial diffusivity (AD), and radial diffusivity (RD), and performed a graph theoretical network analysis to measure global structural connectivity.

**Results:**

A total of 109 patients with focal epilepsy and 88 healthy controls were analyzed. There were no significant differences in the DTI‐ALPS index (1.67 vs. 1.68, *p *= 0.861) between the groups. However, statistically significant associations were found between the DTI‐ALPS index and age (*r* = ‐0.242, *p *= 0.01), FA (*r* = 0.257, *p *= 0.007), MD (*r* = −0.469, *p *< 0.001), AD (*r* = −0.303, *p *= 0.001), RD (*r* = −0.434, *p *< 0.001), and the assortative coefficient (*r* = 0.230, *p *= 0.016) in patients with focal epilepsy.

**Conclusion:**

The main finding of this study is that DTI‐ALPS index is significantly correlated with global DTI parameters and structural connectivity measures of the brain in patients with focal epilepsy. In addition, DTI‐ALPS index decreases with age in these patients. We conclude that the DTI‐ALPS index can be used to investigate glymphatic system function in patients with focal epilepsy.

## Graphical abstract

DTI‐ALPS index is correlated with the global DTI parameters and structural connectivity of the brain in focal epilepsy.

DTI‐ALPS index decreases with age in focal epilepsy patients.

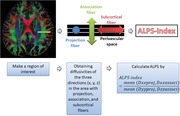

## 1 INTRODUCTION

The glymphatic system is a waste clearance system in the brain that plays an important role in body homeostasis.(Benveniste et al., 2019; Mestre et al., 2020; Rasmussen et al., 2018) It consists of pathways where the subarachnoid cerebrospinal fluid (CSF) enters into the brain parenchyma through the periarterial spaces, blends with parenchymal interstitial fluid and waste products, facilitated by aquaporin‐4 water channels that are embedded in the astrocytic end‐feet, and consecutively drains through the perivenous spaces surrounding veins.(Benveniste et al., 2019; Mestre et al., 2020; Rasmussen et al., 2018) Dysfunction of the glymphatic system has recently been discovered in various neurological diseases, including Alzheimer's dementia, traumatic brain injury, multiple sclerosis, idiopathic normal pressure hydrocephalus, and small vessel disease.(Benveniste et al., 2019; Mestre et al., 2020; Rasmussen et al., 2018)

Epilepsy is one of the most common neurological diseases (Sadr et al., 2018). So far, there have been only a few studies investigating the potential role of the glymphatic system in the pathogenesis of epilepsy (Liu et al., 2020; Salimeen et al., 2021). One recent study analyzed glymphatic system function in patients with febrile seizures and epilepsy using Virchow–Robin space counts and volume and demonstrated that febrile seizures are associated with glymphatic system dysfunction (Salimeen et al., 2021). Another study in patients with idiopathic generalized epilepsy using the same methods established that epileptic seizures may also change glymphatic system function (Liu et al., 2020). It is plausible that the impairment of the blood‐brain barrier related to a dysfunction of the endothelial tight junctions caused by proinflammatory mediators in epilepsy contributes to an abnormal exchange between CSF and interstitial fluid, thus affecting the glymphatic system (Rabinovitch et al., 2019; Rabinovitch, Aviram et al., 2020; Vezzani et al., 2013). Considering that the glymphatic system is most active during sleep and many patients with epilepsy have poor sleep quality, this may further alter glymphatic system function (Anzai & Minoshima, 2021). To date, no studies have investigated glymphatic system function in patients with focal epilepsy.

There are a variety of methods to assess the function of the glymphatic system in humans (Taoka & Naganawa, 2020a, 2020b). Among these, diffusion tensor imaging analysis along the perivascular space (DTI‐ALPS) is particularly useful in clinical practice because it does not require intrathecal injection by lumbar puncture or gadolinium‐based contrast agents deposition in the brain (Taoka et al., 2017). Although DTI‐ALPS index is reported to be influenced by the imaging plane, the number of motion‐proving gradients, and echo time in the imaging sequence, the index is robust under the fixed imaging method (Taoka et al., 2021). At this point, no studies have utilized DTI‐ALPS index to assess glymphatic system function in epilepsy.

DTI maps and characterizes the three‐dimensional diffusion of water as a function of spatial location (Sundgren et al., 2004). Water diffusion within tissues is altered by impaired microstructure integrity of white matter. Consequently, DTI is a powerful tool for characterizing the effects of diseases on tissue microstructure by measuring the global DTI parameters, including the fractional anisotropy (FA), mean diffusivity (MD), axial diffusivity (AD), and radial diffusivity (RD; Sundgren et al., 2004). In addition, DTI has recently been used to evaluate structural connectivity and brain networks and shown good test‐retest reliability of graph theory measures of structural connectivity (Dennis et al., 2012).

In this study, we aimed to investigate the DTI‐ALPS index in patients with newly diagnosed focal epilepsy and normal brain magnetic resonance imaging (MRI) and to compare it to that of healthy controls, thereby eliminating potential effects of anti‐seizure medication (ASM). Furthermore, we sought to use correlation analysis to understand the relationship between DTI‐ALPS index and clinical characteristics, global DTI parameters, and global structural connectivity. We hypothesized that there would be significant associations between these factors in patients with focal epilepsy. These confirmations will contribute towards discovering the pathogenesis of epilepsy.

## 2 METHODS

### 2.1 Participants

This was a retrospective study conducted at a single epilepsy center.

We included patients with epilepsy based on the following eligibility criteria: (Berg et al., 2010) 1) ictal semiology and electroencephalography findings compatible with focal epilepsy; 2) newly diagnosed epilepsy with drug‐naïve status; 3) having undergone DTI between March 2018 and March 2021; 4) sufficient quality of DTI for quantitative analysis; 5) no structural lesions on routine brain MRI in the visual assessment; and 6) no further medical, neurological, or psychiatric diseases.

We obtained the demographic and clinical characteristics of patients, such as age, sex, age at seizure onset, time from the first seizure until the performance of MRI, and seizure frequency (total number of seizures before MRI taken) from the hospital's electronic medical records.

As a control group, we included age‐ and sex‐matched healthy controls from one of our previous studies (Jang et al., 2017). All controls had a normal brain MRI based on visual inspection and no medical, neurological, or psychiatric diseases.

The study protocol was approved by the institutional review body of our hospital. Informed written consent was waived because of the retrospective nature of the study.

### 2.2 DTI acquisition

All MRI scans of the patients with focal epilepsy and healthy controls had been obtained using the same 3T MRI scanner with a 32‐channel head coil (Achieva 3.0 TX, Philips Healthcare). In our epilepsy patients, these had been obtained as part of their routine work‐up. DTI was conducted using spin‐echo single‐shot echo‐planar pulse sequences with a total of 32 different diffusion directions (repetition time/echo time = 8620/85 ms, flip angle = 90 °, slice thickness = 2.25 mm, acquisition matrix = 120 × 120, field of view = 240 × 240 mm^2^, and *b*‐value = 1000 s/mm^2^).

### 2.3 DTI processing

We processed the DTI data using the DSI Studio software, version 2021 May (http://dsi‐studio.labsolver.org). We read the DTI raw files in the Digital Imaging and Communications in Medicine standard format. Then, we did correct the eddy current and phase distortion artifact. We set up a mask to filter out the background region, increase reconstruction efficacy, and facilitate further visualization. We performed reconstruction applying the DTI method to characterize the major direction of water diffusion and subsequently fiber tracking using default parameters. Whole‐brain seeding was conducted with a total of 10,000 seeds, and the angular threshold was 60 °. Tracks of less than 30 mm length were discarded. The automated anatomical labeling template was used for brain parcellation, and every white matter fiber was evaluated for extreme points.

### 2.4 Obtaining the measures of global DTI parameters and global structural connectivity

Consecutively, we calculated the measures of global DTI parameters using region statistics in DSI studio based on whole‐brain seeding, including whole‐brain FA, MD, AD, and RD. In the next step, we generated a connectivity matrix for each subjects, using the number of tracts that passed the two region‐of‐interest (ROI) threshold by 0.001 of the sum to obtain the network measures. Graph theoretical analysis views brain connections as a graph and applied graph‐based measures to analyze it. A graph is defined as a set of nodes or vertices and the edges or lines between them. We used built‐in atlas provided in DSI studio, and we conducted a spatial normalization with the linear transformation. Finally, we performed a graph theoretical network analysis and calculated the measures of global structural connectivity from the connectivity matrix at fixed density, including the assortative coefficient, mean clustering coefficient, characteristic path length, diameter, radius, global efficiency, local efficiency, small‐worldness index, and transitivity. We used the weighted network measures in which the connectivity matrix is normalized so that the maximum value of the matrix is one.

### 2.5 Calculation of DTI‐ALPS index

We drew a rectangular ROI and obtained the fiber orientation and diffusivities in all three directions along the x‐, y‐, and z‐axes as voxel levels. Among the several voxels, we selected one voxel for each fiber on the same x‐axis (projection, association, and subcortical fibers), which showed the most frequent orientation in each fiber. The DTI‐ALPS index was calculated using the following formula:(Taoka et al., 2017)

$$ALPS\;index\;=\frac{mean\;\left(Dxxproj,\;Dxxassoci\right)}{mean\;\left(Dyyproj,Dzzassoci\right)}\;$$

Dxxproj: diffusivity along the x‐axis in the projection fiber, Dxxassoci: diffusivity along the x‐axis in the association fiber, Dyyproj: diffusivity along the y‐axis in the projection fiber, Dzzassoci: diffusivity along the z‐axis in the association fiber.

**FIGURE 1:**
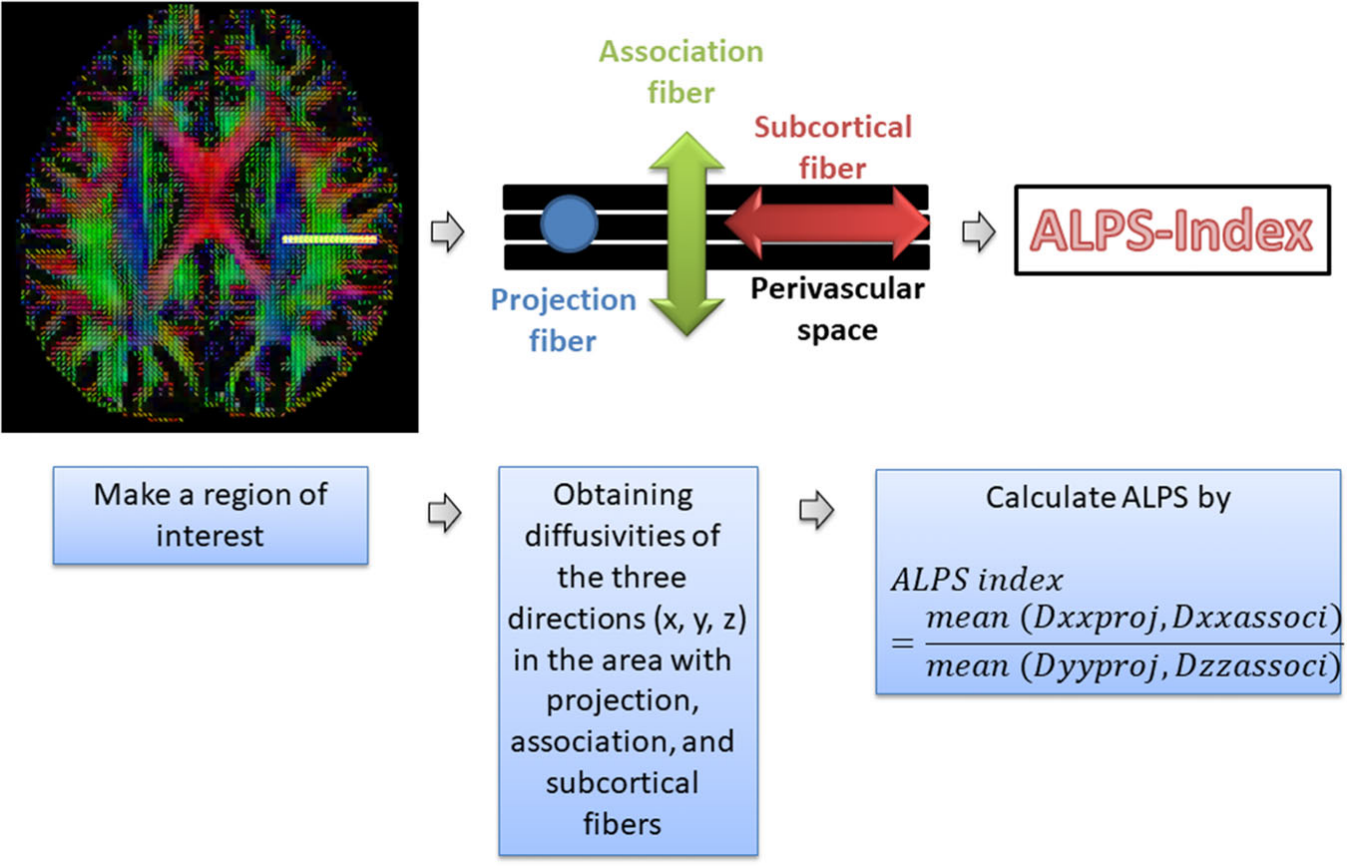
Calculation of diffusion tensor imaging analysis along the perivascular space index. Figure is generated in the courtesy of our previous study (H. J. Lee et al., 2021).

Figure 1 showed the process for calculation of DTI‐ALPS index in this study.

### 2.6 Statistical analysis

Categorical variables were expressed as numbers and percentages. Continuous variables with normal distribution were presented as the mean with standard deviations and continuous variables without normal distribution as the median values with interquartile ranges. Statistical comparisons were conducted using the chi‐square test for categorical variables and the independent samples t‐test for continuous variables. Correlation analysis was conducted with Pearson's correlation test. Statistical significance was defined as a two‐tailed *p*‐value of < 0.05.

We performed the Bonferroni correction for the multiple comparisons of the measures of global DTI parameters and global structural connectivity (global DTI parameters, *p *< 0.012 [0.05/4 values]; structural connectivity, *p *< 0.005 [0.05/9 network measures]) between the groups.

All statistical analyses were performed using MedCalc® Statistical Software, version 20 (MedCalc Software Ltd.; https://www.medcalc.org; 2021).

## 3 DATA AVAILABILITY STATEMENT

The data in this study are available from the corresponding author upon reasonable request.

## 4 RESULTS

### 4.1 Clinical characteristics of the participants

One hundred and nine patients with focal epilepsy and 88 healthy controls were enrolled in the study. Table 1 shows the clinical characteristics compared between the groups. The mean age and sex distribution were not statistically significantly different between patients with focal epilepsy and the healthy controls (38.2 vs. 41.5 years, *p *= 0.20; males 58 [53.2%] and 47 [53.4%], *p *= 0.98, respectively).

**TABLE 1:** Clinical characteristics of the participants in this study on the glymphatic system in focal epilepsy

	Patients with focal epilepsy (n = 109)	Healthy controls (n = 88)	*p*‐Value
Age (mean±SD), years	38.2±19.0	41.5±16.6	0.20
Male, n (%)	58 (53.2)	47 (53.4)	0.98
Age of onset, years	35.5 (19−52)		
Time between the first seizure and MRI, days	105 (7−1003)		
Seizures before MRI, n	2 (2−3)		

Data are presented as the median (interquartile range) unless indicated otherwise.

SD, standard deviation; MRI, magnetic resonance imaging.

### 4.2 Differences in the global DTI parameters between patients with focal epilepsy and healthy controls

Table 2 compares global DTI parameters between patients with focal epilepsy and healthy controls. The AD value was significantly higher in patients with focal epilepsy than in healthy controls (1.21 vs. 1.19, *p *= 0.003), whereas the FA, MD, and RD did not differ statistically significantly after the Bonferroni correction between them.

**TABLE 2:** Differences in the global DTI parameters between patients with focal epilepsy and healthy controls in this study on the glymphatic system

Global DTI parameters	Patients with focal epilepsy	Healthy controls	
	(n = 109)	(n = 88)	*p*‐Value
Fractional anisotropy	0.352±0.048	0.339±0.012	0.04
Mean diffusivity	0.882±0.053	0.874±0.026	0.53
Axial diffusivity	1.212±0.055	1.190±0.027	*0.003
Radial diffusivity	0.716±0.067	0.720±0.026	0.64

* *p *< 0.012.

### 4.3 The differences in the global structural connectivity between patients with focal epilepsy and healthy controls

There were no statistically significant differences in the measures of global structural connectivity (assortative coefficient, mean clustering coefficient, characteristic path length, diameter, radius, global efficiency, local efficiency, small‐worldness index, and transitivity) between the groups (Table 3).

**TABLE 3:** Differences in global structural connectivity between patients with focal epilepsy and healthy controls in this study on the glymphatic system

Structural connectivity measures	Patients with focal epilepsy	Healthy controls	
	(n = 109)	(n = 88)	*p*‐Value
Mean clustering coefficient	0.145±0.088	0.140±0.082	0.74
Global efficiency	1.053±0.273	1.075±0.307	0.63
Local efficiency	1.444±0.558	1.517±0.621	0.44
Characteristic path length	4.072±0.423	4.210±0.542	0.07
Small‐worldness index	0.124±0.109	0.122±0.101	0.90
Transitivity	0.159±0.107	0.159±0.101	0.99
Radius	2.024±0.337	1.996±0.342	0.60
Diameter	3.822±0.607	3.760±0.528	0.50
Assortative coefficient	0.124±0.090	0.123±0.094	0.95

### 4.4 The differences in DTI‐ALPS index between patients with focal epilepsy and healthy controls

**FIGURE 2:**
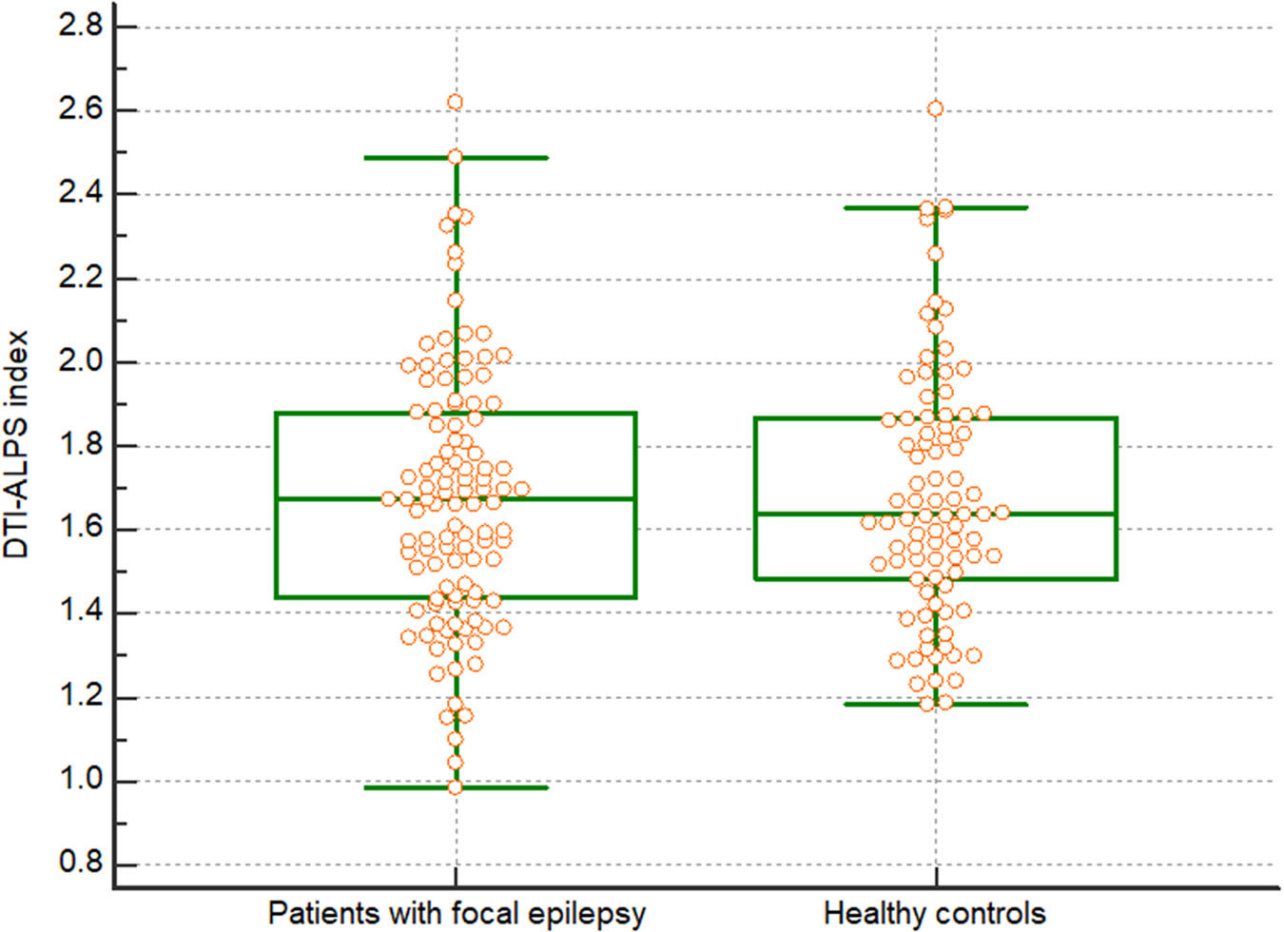
Diffusion tensor imaging analysis along the perivascular space index for patients with focal epilepsy (n = 109) and healthy controls (n = 88) in this study on the glymphatic system. No significant differences in the diffusion tensor imaging analysis along the perivascular space indices existed between the groups

No statistically significant differences in the DTI‐ALPS index were observed between patients with focal epilepsy and healthy controls (1.67 vs. 1.68, *p *= 0.86) (Figure 2).

### 4.5 Correlation between variables and DTI‐ALPS index in patients with focal epilepsy

**FIGURE 3:**
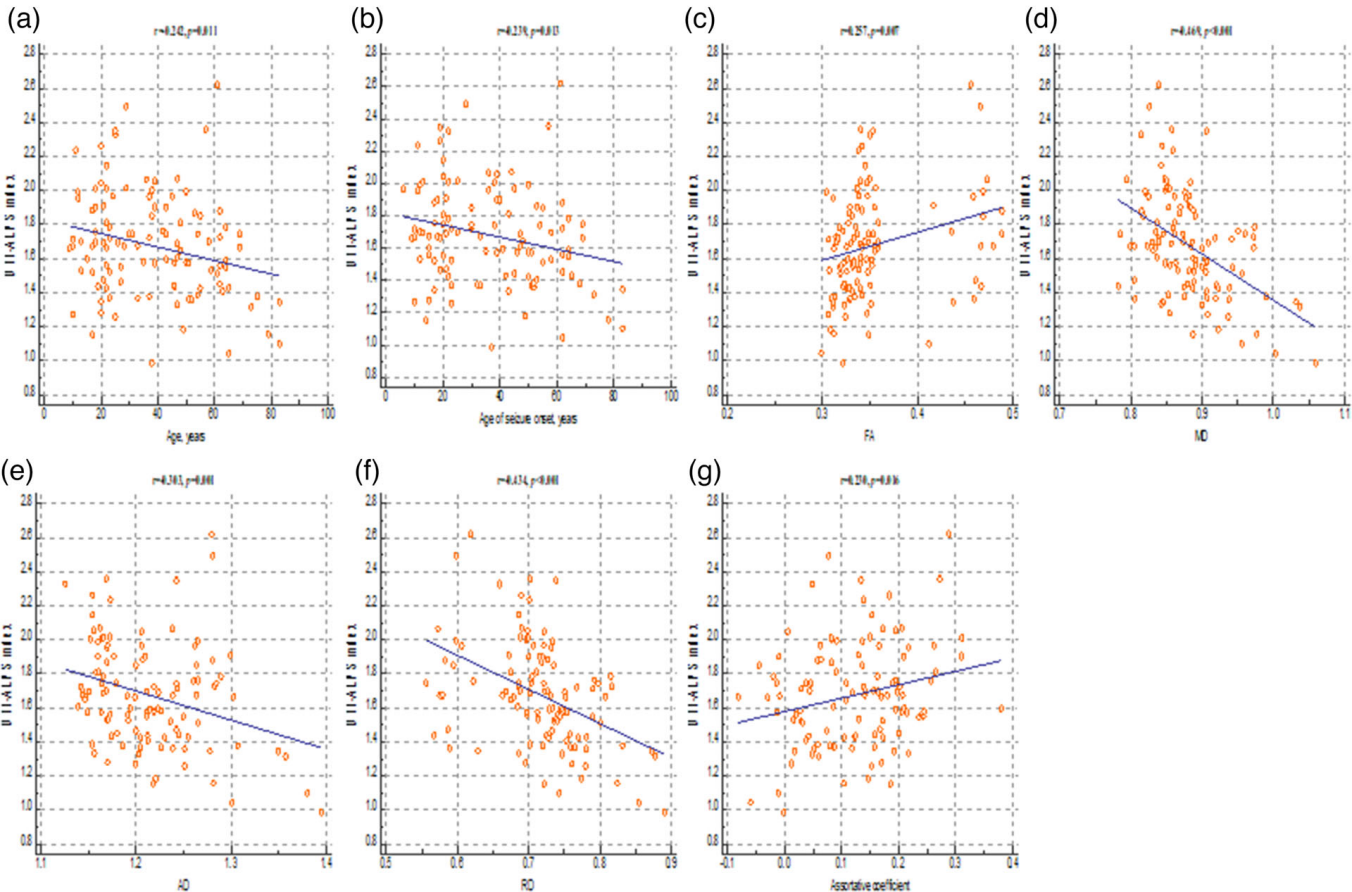
Correlation analysis between the index of diffusion tensor imaging analysis along the perivascular space and the clinical characteristics, global DTI parameters, and measures of structural connectivity in patients with focal epilepsy. These figures reveal a significant positive correlation between the diffusion tensor imaging analysis along the perivascular space index, fractional anisotropy value (C), and assortative coefficient (G), whereas a negative correlation was found between the diffusion tensor imaging analysis along the perivascular space index and age (A), age at seizure onset (B), mean diffusivity (D); axial diffusivity (E), and radial diffusivity values (F). FA, fractional anisotropy; MD, mean diffusivity; AD, axial diffusivity; RD, radial diffusivity

Significant correlations were observed between the DTI‐ALPS index and age (r = −0.242, *p *= 0.01), age at seizure onset (r = −0.239, *p *= 0.01), FA (r = 0.257, *p *= 0.007), MD (r = −0.469, *p *< 0.001), AD (r = −0.303, *p *= 0.001), RD (r = −0.434, *p *< 0.001), and the assortative coefficient (r = 0.230, *p *= 0.02) in patients with focal epilepsy (Figure 3).

However, other measures of clinical characteristics, including duration of seizures (r = 0.002, *p *= 0.98) and seizure frequency (r = −0.026, *p *= 0.79), and the measure of structural connectivity, including mean clustering coefficient (r = −0.050, *p *= 0.61), characteristic path length (r = −0.013, *p *= 0.89), diameter (r = 0.012, *p *= 0.90), radius (r = 0.071, *p *= 0.46), global efficiency (r = 0.101, *p *= 0.30), local efficiency (r = 0.046, *p *= 0.63), small‐worldness index (r = −0.018, *p *= 0.85), and transitivity (r = −0.010, *p *= 0.92), were not significantly correlated with the DTI‐ALPS index.

### 4.6 Correlation between variables and DTI‐ALPS index in healthy controls

Significant correlations were also observed between the DTI‐ALPS index and age (r = −0.226, *p *= 0.02), FA (r = 0.198, *p *= 0.01), MD (r = −0.369, *p *< 0.001), AD (r = −0.245, *p *= 0.002), RD (r = −0.344, *p *= 0.001), and the assortative coefficient (r = 0.183, *p *= 0.026) in healthy controls.

However, other measures of structural connectivity, including mean clustering coefficient (r = −0.001, *p *= 0.99), characteristic path length (r = 0.018, *p *= 0.81), diameter (r = 0.010, *p *= 0.89), radius (r = 0.047, *p *= 0.54), global efficiency (r = 0.077, *p *= 0.31), local efficiency (r = 0.062, *p *= 0.42), small‐worldness index (r = 0.027, *p *= 0.72), and transitivity (r = 0.026, *p *= 0.74), were not significantly correlated with the DTI‐ALPS index.

## 5 DISCUSSION

The main findings of this study on the glymphatic system in newly diagnosed patients with focal epilepsy with normal brain MRI were that 1) the DTI‐ALPS index was not statistically significantly different between these patients and healthy controls, suggesting that there was no difference in glymphatic system function; 2) negative correlations existed between the DTI‐ALPS index and subjects’ age, indicating that their glymphatic system function decreased with aging, and 3) statistically significant correlations existed between the DTI‐ALPS index and the FA, MD, AD, RD, and assortative coefficient, demonstrating that the glymphatic system function was correlated with global DTI parameters and structural connectivity. These findings confirmed our hypothesis.

The strengths of our study were: 1) It was the first to evaluate the DTI‐ALPS index in patients with focal epilepsy; 2) We only analyzed patients with newly diagnosed focal epilepsy and a normal brain MRI, increasing the homogeneity of patients and excluding potential effects of ASM on DTI‐ALPS index; and 3) We enrolled large patients with focal epilepsy, which was possible because of the specific routine epilepsy MRI protocols at our center that include DTI.

Regarding our finding that DTI‐ALPS index decreased with age in both patients with focal epilepsy and healthy subjects, a previous study found that age was related to a steep decline in glymphatic system function in the brain of wild‐type mice (Kress et al., 2014). Advanced age was associated with a dramatic reduction in the efficiency of the exchange between the subarachnoid CSF and the brain parenchyma, evaluated by fluorescence microscopy and radiotracer clearance assays (Kress et al., 2014). In healthy Chinese subjects, arterial stiffening and compliance reductions progressed with aging, which was interpreted by the authors as being caused by a degeneration of the elastic lamellar architecture of the arterial wall (Avolio et al., 1983). In addition, mechanical remodeling of the microvasculature, resulting from the deposition of advanced glycation end‐products in the vessel wall and intima and media calcification, contributes to arterial stiffening seen with aging (O'Rourke & Safar, 2005). Worsening vessel stiffness increases arterial pulse wave velocity and pressure in the brain, which affects the pulsatile component of cerebral blood flow and CSF transport (Tsao et al., 2013). These observations may explain why glymphatic system function decreases with age.

We found that whole‐brain FA was positively and MD was negatively correlated with the DTI‐ALPS index, which corresponds to previous results. FA reflects the main direction of water diffusion within a given voxel and MD is the rate of water diffusion, irrespective of its direction (Assaf & Pasternak, 2008). A higher FA is linked to preserved fiber integrity, whereas increases in MD are related to structural disintegration (Assaf & Pasternak, 2008). While AD is related to axonal integrity, RD provides information about the degree of myelination (Winklewski et al., 2018). Both were negatively correlated with the DTI‐ALPS index in our patients. We interpret the negative correlation in FA and concomitant positive correlation in MD, AD, and RD as potential signs of global DTI parameters associated with poor glymphatic system function in our patients. A previous study examined the relationships between whole‐brain FA, MD, AD, RD, and composite scores of memory and executive function and found significant relationships between the FA, MD, AD, and RD and executive function, suggesting that DTI has the potential to measure cognitive function (Mayo et al., 2018). Interestingly, an earlier study using the DTI‐ALPS method also showed that the glymphatic system function correlated with cognitive function (Zhang et al., 2021). Thus, we can assume that glymphatic system function is related to global DTI parameters and cognitive function.

Our study showed a positive correlation between the DTI‐ALPS index and assortative coefficient as one of the measures reflecting global structural connectivity. Increasing evidence exists that focal epilepsy is a network disease, and DTI is used to assess structural brain connectivity (D. A. Lee et al., 2021; van Diessen et al., 2014). This study is the first to demonstrate a significant association between brain connectivity and glymphatic system function. The coefficient measures assortativity, meaning the tendency of nodes with similar properties to connect with each other (Newman, 2002). There were reports that epileptic networks show more assortative patterns (Bialonski & Lehnertz, 2013; Sone et al., 2016). We previously demonstrated that the assortative coefficient predicted ASM response in patients with newly diagnosed focal epilepsy (Park et al., 2020). Further studies investigating the relationship between glymphatic system function and brain connectivity are needed to scrutinize our findings.

However, we could not find any differences in the DTI‐ALPS index between patients with focal epilepsy and healthy controls, suggesting no alterations in glymphatic system function in focal epilepsy. This finding was inconsistent with previous reports showing glymphatic system dysfunction in patients with epilepsy (Liu et al., 2020; Salimeen et al., 2021). This discrepancy may originate from several factors. We only investigated patients with newly diagnosed epilepsy who were drug‐naïve to exclude potential effects of ASM on glymphatic system function. Furthermore, we only included patients with focal epilepsy and a normal brain MRI, which were different criteria from those in previous studies. Finally, we investigated glymphatic system function with DTI‐ALPS, whereas previous studies used Virchow‐Robin space counts and volume (Liu et al., 2020; Salimeen et al., 2021).

Only a few studies have used DTI‐ALPS to assess the glymphatic system function in neurological diseases, i.e., Alzheimer's disease (Taoka et al., 2017), Parkinson's disease (Chen et al., 2021), cerebral small vessel disease (Zhang et al., 2021), and idiopathic normal pressure hydrocephalus (Bae et al., 2021). All of these studies demonstrated glymphatic system dysfunction in these conditions. Our study also confirmed that the DTI‐ALPS index could be used to investigate glymphatic system function in patients with epilepsy, providing a foundation for future studies.

There are several limitations to this study. First, this study was retrospectively conducted at a single epilepsy center. Multicenter studies with larger sample sizes are required to confirm our findings. Second, this was a retrospective study. Follow‐up data to investigate the DTI‐ALPS index in patients with focal epilepsy are needed to determine the causal relationship among DTI‐ALPS index, global DTI parameters, and structural connectivity. Third, although we only analyzed patients with focal epilepsy and normal brain MRI, the origins of epileptic seizures varied.

## 6 CONCLUSION

The main findings of this study are that DTI‐ALPS index is significantly correlated with global DTI parameters and structural connectivity and decreases with age in patients with focal epilepsy. However, the DTI‐ALPS index in newly diagnosed focal epilepsy patients with normal brain MRI does not differ from that in healthy controls. The DTI‐ALPS index can be used to investigate glymphatic system function in patients with focal epilepsy.

## CONFLICT OF INTEREST

All authors have no conflicts of interest to declare at the time of submission.

## AUTHOR CONTRIBUTIONS

DA Lee, BS Park, and KM Park were associated with conception and design. BS Park, J Ko, S Park, JH Park, IH Kim, YJ Lee, and KM Park were associated with the acquisition of data, analysis, and interpretation of data. DA Lee, BS Park, and KM Park drafted and revised the manuscript. KM Park provided the final approval.

## FUNDING

None.

### PEER REVIEW

The peer review history for this article is available at https://publons.com/publon/10.1002/brb3.2504

## Data Availability

The data that support the findings of this study are available from the corresponding author upon reasonable request.

## References

[brb32504-bib-0001] Anzai, Y. , & Minoshima, S. (2021). Why we need to sleep: Glymphatic pathway and neurodegenerative disease. Radiology, 300, 211140. 10.1148/radiol.2021211140 34156305

[brb32504-bib-0002] Assaf, Y. , & Pasternak, O. (2008). Diffusion tensor imaging (DTI)‐based white matter mapping in brain research: A review. Journal of Molecular Neuroscience, 34(1), 51–61. 10.1007/s12031-007-0029-0 18157658

[brb32504-bib-0003] Avolio, A. P. , Chen, S. G. , Wang, R. P. , Zhang, C. L. , Li, M. F. , & O'Rourke, M. F. (1983). Effects of aging on changing arterial compliance and left ventricular load in a northern Chinese urban community. Circulation, 68(1), 50–58. 10.1161/01.cir.68.1.50 6851054

[brb32504-bib-0004] Bae, Y. J. , Choi, B. S. , Kim, J. M. , Choi, J. H. , Cho, S. J. , & Kim, J. H. (2021). Altered glymphatic system in idiopathic normal pressure hydrocephalus. Parkinsonism & Related Disorders, 82, 56–60. 10.1016/j.parkreldis.2020.11.009 33248394

[brb32504-bib-0005] Benveniste, H. , Liu, X. , Koundal, S. , Sanggaard, S. , Lee, H. , & Wardlaw, J. (2019). The glymphatic system and waste clearance with brain aging: A review. Gerontology, 65(2), 106–119. 10.1159/000490349 29996134 PMC6329683

[brb32504-bib-0006] Berg, A. T. , Berkovic, S. F. , Brodie, M. J. , Buchhalter, J. , Cross, J. H. , Van Emde Boas, W. , Engel, J. , French, J. , Glauser, T. A. , Mathern, G. W. , Moshé, S. L. , Nordli, D. , Plouin, P. , & Scheffer, I. E. (2010). Revised terminology and concepts for organization of seizures and epilepsies: Report of the ILAE Commission on classification and terminology, 2005–2009. Epilepsia, 51(4), 676–685. 10.1111/j.1528-1167.2010.02522.x 20196795

[brb32504-bib-0007] Bialonski, S. , & Lehnertz, K. (2013). Assortative mixing in functional brain networks during epileptic seizures. Chaos (Woodbury, N.Y.), 23(3), 033139. 10.1063/1.4821915 24089975

[brb32504-bib-0008] Chen, H.‐L., Chen, P.‐C. , Lu, C.‐H. , Tsai, N.‐W. , Yu, C.‐C. , Chou, K.‐H. , Lai, Y.‐R. , Taoka, T. , & Lin, W.‐C. (2021). Associations among cognitive functions, plasma DNA, and diffusion tensor image along the perivascular space (DTI‐ALPS) in patients with Parkinson's disease. Oxid Med Cell Longev, 2021, 4034509. 10.1155/2021/4034509 33680283 PMC7904342

[brb32504-bib-0009] Dennis, E. L. , Jahanshad, N. , Toga, A. W. , McMahon, K. L. , de Zubicaray, G. I. , Martin, N. G. , Wright, M. J. , & Thompson, P. M. (2012). Test‐retest reliability of graph theory measures of structural brain connectivity. Med Image Comput Comput Assist Interv, 15(Pt 3), 305–312. 10.1007/978-3-642-33454-2_38 PMC4039303 23286144

[brb32504-bib-0010] Jang, H. , Lee, J. Y. , Lee, K. I. , & Park, K. M. (2017). Are there differences in brain morphology according to handedness? Brain Behav, 7(7), e00730. 10.1002/brb3.730 28729936 PMC5516604

[brb32504-bib-0011] Kress, B. T. , Iliff, J. J. , Xia, M. , Wang, M. , Wei, H. S. , Zeppenfeld, D. , Xie, L. , Kang, H. , Xu, Q. , Liew, J. A. , Plog, B. A. , Ding, F. , Deane, R. , & Nedergaard, M. (2014). Impairment of paravascular clearance pathways in the aging brain. Annals of Neurology, 76(6), 845–861. 10.1002/ana.24271 25204284 PMC4245362

[brb32504-bib-0012] Lee, D. A. , Lee, H. J. , Kim, H. C. , & Park, K. M. (2021). Temporal lobe epilepsy with or without hippocampal sclerosis: Structural and functional connectivity using advanced MRI techniques. Journal of Neuroimaging, 31(5), 973–980. 10.1111/jon.12898 34110654

[brb32504-bib-0013] Lee, H. J. , Lee, D. A. , Shin, K. J. , & Park, K. M. (2021). Glymphatic system dysfunction in patients with juvenile myoclonic epilepsy. Journal of Neurology, 10.1007/s00415-021-10799-w 34510256

[brb32504-bib-0014] Liu, C. , Habib, T. , Salimeen, M. , Pradhan, A. , Singh, M. , Wang, M. , Wu, F. , Zhang, Y. , Gao, L. , Yang, G. , Li, X. , & Yang, J. (2020). Quantification of visible Virchow‐Robin spaces for detecting the functional status of the glymphatic system in children with newly diagnosed idiopathic generalized epilepsy. Seizure: The Journal of the British Epilepsy Association, 78, 12–17. 10.1016/j.seizure.2020.02.015 32151968

[brb32504-bib-0015] Mayo, C. D. , Garcia‐Barrera, M. A. , Mazerolle, E. L. , Ritchie, L. J. , Fisk, J. D. , Gawryluk, J. R. , & Alzheimer's Disease Neuroimaging, I. (2018). Relationship between DTI metrics and cognitive function in Alzheimer's disease. Front Aging Neurosci, 10, 436. 10.3389/fnagi.2018.00436 30687081 PMC6333848

[brb32504-bib-0016] Mestre, H. , Mori, Y. , & Nedergaard, M. (2020). The brain's glymphatic system: Current controversies. Trends in Neuroscience (Tins), 43(7), 458–466. 10.1016/j.tins.2020.04.003 PMC7331945 32423764

[brb32504-bib-0017] Newman, M. E. (2002). Assortative mixing in networks. Physical Review Letter, 89(20), 208701. 10.1103/PhysRevLett.89.208701 12443515

[brb32504-bib-0018] O'Rourke, M. F. , & Safar, M. E. (2005). Relationship between aortic stiffening and microvascular disease in brain and kidney: Cause and logic of therapy. Hypertension, 46(1), 200–204. 10.1161/01.HYP.0000168052.00426.65 15911742

[brb32504-bib-0019] Park, K. M. , Cho, K. H. , Lee, H. J. , Heo, K. , Lee, B. I. , & Kim, S. E. (2020). Predicting the antiepileptic drug response by brain connectivity in newly diagnosed focal epilepsy. Journal of Neurology, 267(4), 1179–1187. 10.1007/s00415-020-09697-4 31925497

[brb32504-bib-0020] Rabinovitch, A. , Aviram, I. , Biton, Y. , & Braunstein, D. (2020). Explaining recent postictal epilepsy EEG results by the G‐lymphatic clearance hypothesis. Medical Hypotheses, 137, 109600. 10.1016/j.mehy.2020.109600 32006922

[brb32504-bib-0021] Rabinovitch, A. , Aviramd, I. , Biton, Y. , & Braunstein, D. (2019). A combined astrocyte ‐ G‐lymphatic model of epilepsy initiation, maintenance and termination. Medical Hypotheses, 133, 109384. 10.1016/j.mehy.2019.109384 31494484

[brb32504-bib-0022] Rasmussen, M. K. , Mestre, H. , & Nedergaard, M. (2018). The glymphatic pathway in neurological disorders. Lancet Neurology, 17(11), 1016–1024. 10.1016/S1474-4422(18)30318-1 30353860 PMC6261373

[brb32504-bib-0023] Sadr, S. S. , Javanbakht, J. , Javidan, A. N. , Ghaffarpour, M. , Khamse, S. , & Naghshband, Z. (2018). Descriptive epidemiology: Prevalence, incidence, sociodemographic factors, socioeconomic domains, and quality of life of epilepsy: An update and systematic review. Arch Med Sci, 14(4), 717–724. 10.5114/aoms.2016.60377 30002687 PMC6040113

[brb32504-bib-0024] Salimeen, M. S. A. , Liu, C. , Li, X. , Wang, M. , Singh, M. , Si, S. , Li, M. , Cheng, Y. , Wang, X. , Zhao, H. , Wu, F. , Zhang, Y. , Tafawa, H. , Pradhan, A. , Yang, G. , & Yang, J. (2021). Exploring variances of white matter integrity and the glymphatic system in simple febrile seizures and epilepsy. Front Neurol, 12, 595647. 10.3389/fneur.2021.595647 33967932 PMC8097149

[brb32504-bib-0025] Sone, D. , Matsuda, H. , Ota, M. , Maikusa, N. , Kimura, Y. , Sumida, K. , Yokoyama, K. , Imabayashi, E. , Watanabe, M. , Watanabe, Y. , Okazaki, M. , & Sato, N. (2016). Impaired cerebral blood flow networks in temporal lobe epilepsy with hippocampal sclerosis: A graph theoretical approach. Epilepsy & Behavior, 62, 239–245. 10.1016/j.yebeh.2016.07.016 27497065

[brb32504-bib-0026] Sundgren, P. C. , Dong, Q. , Gomez‐Hassan, D. , Mukherji, S. K. , Maly, P. , & Welsh, R. (2004). Diffusion tensor imaging of the brain: Review of clinical applications. Neuroradiology, 46(5), 339–350. 10.1007/s00234-003-1114-x 15103435

[brb32504-bib-0027] Taoka, T. , Ito, R. , Nakamichi, R. , Kamagata, K. , Sakai, M. , Kawai, H. , Nakane, T. , Abe, T. , Ichikawa, K. , Kikuta, J. , Aoki, S. , & Naganawa, S. (2021). Reproducibility of diffusion tensor image analysis along the perivascular space (DTI‐ALPS) for evaluating interstitial fluid diffusivity and glymphatic function: Changes in Alps index on multiple condition acquisition experiment (CHAMONIX) study. Japanese Journal of Radiology, 10.1007/s11604-021-01187-5 PMC8803717 34390452

[brb32504-bib-0028] Taoka, T. , Masutani, Y. , Kawai, H. , Nakane, T. , Matsuoka, K. , Yasuno, F. , Kishimoto, T. , & Naganawa, S., (2017). Evaluation of glymphatic system activity with the diffusion MR technique: Diffusion tensor image analysis along the perivascular space (DTI‐ALPS) in Alzheimer's disease cases. Jpn J Radiol, 35(4), 172–178. 10.1007/s11604-017-0617-z 28197821

[brb32504-bib-0029] Taoka, T. , & Naganawa, S. (2020a). Glymphatic imaging using MRI. Journal of Magnetic Resonance Imaging, 51(1), 11–24. 10.1002/jmri.26892 31423710

[brb32504-bib-0030] Taoka, T. , & Naganawa, S. (2020b). Neurofluid dynamics and the glymphatic system: A neuroimaging perspective. Korean Journal of Radiology, 21(11), 1199–1209. 10.3348/kjr.2020.0042 32783417 PMC7462760

[brb32504-bib-0031] Tsao, C. W. , Seshadri, S. , Beiser, A. S. , Westwood, A. J. , Decarli, C. , Au, R. , Himali, J. J. , Hamburg, N. M. , Vita, J. A. , Levy, D. , Larson, M. G. , Benjamin, E. J. , Wolf, P. A. , Vasan, R. S. , & Mitchell, G. F. (2013). Relations of arterial stiffness and endothelial function to brain aging in the community. Neurology, 81(11), 984–991. 10.1212/WNL.0b013e3182a43e1c 23935179 PMC3888200

[brb32504-bib-0032] van Diessen, E. , Zweiphenning, W. J. , Jansen, F. E. , Stam, C. J. , Braun, K. P. , & Otte, W. M. (2014). Brain network organization in focal epilepsy: A systematic review and meta‐analysis. Plos One, 9(12), e114606. 10.1371/journal.pone.0114606 25493432 PMC4262431

[brb32504-bib-0033] Vezzani, A. , Aronica, E. , Mazarati, A. , & Pittman, Q. J. (2013). Epilepsy and brain inflammation. Experimental Neurology, 244, 11–21. 10.1016/j.expneurol.2011.09.033 21985866

[brb32504-bib-0034] Winklewski, P. J. , Sabisz, A. , Naumczyk, P. , Jodzio, K. , Szurowska, E. , & Szarmach, A. (2018). Understanding the physiopathology behind axial and radial diffusivity changes‐What do we know? Front Neurol, 9, 92. 10.3389/fneur.2018.00092 29535676 PMC5835085

[brb32504-bib-0035] Zhang, W. , Zhou, Y. , Wang, J. , Gong, X. , Chen, Z. , Zhang, X. , Cai, J. , Chen, S. , Fang, L. , Sun, J. , & Lou, M. (2021). Glymphatic clearance function in patients with cerebral small vessel disease. Neuroimage, 238, 118257. 10.1016/j.neuroimage.2021.118257 34118396

